# Recurrent spontaneous intracerebral hemorrhage associated with polyglobulia

**DOI:** 10.11604/pamj.2015.22.128.6486

**Published:** 2015-10-13

**Authors:** Salami Mohcine, El Mostarchid Brahim

**Affiliations:** 1 Service de Neurochirurgie de l'Hôpital Militaire d'Instruction Mohammed V, Rabat, Maroc

**Keywords:** Recurrent spontaneous intracerebral hemorrhage, polyglobulia, prognosis

## Image in medicine

Spontaneous intracerebral hemorrhage (ICH) accounts for 15% of stroke cases in the US and Europe and up to 30% in Asian populations. Intracerebral hemorrhage is a relatively uncommon form of stroke-it causes only 10 to 15 percent of all strokes. It is more disabling and has a higher mortality rate than ischemic stroke, and it can occur at any age. It is slightly more common in men than in women. Its etiologies are dominated by hypertension, arteriovenous malformation, aneurysmal rupture, cerebral amyloid angiopathy, intracranial neoplasm and coagulopathy. We report the first case to date of spontaneous intracerebral hemorrhage associated with polyglobulia. A 56-years-old man, with history of polyglobulia and spontaneous intracerebral hemorrhage treated surgically in 2011, was admitted to the emergency service with the complaint of headache, disturbance of consciousness. Clinical examination is estimated GCS 13/15 and 2/5 left hemiparesis. An emergency CT-scan revealed a recurrent parieto-temporal hematoma, sequelar frontal hypodensity of the old stroke with its cranial flap.

**Figure 1:**
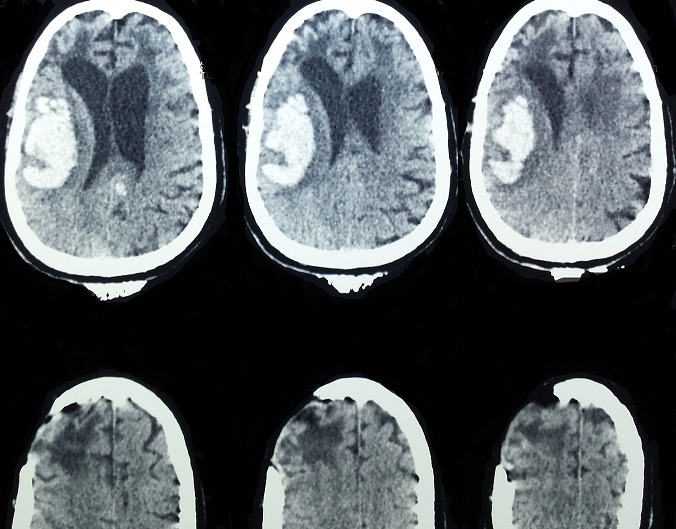
Cranial CT-scan showing an acute right parieto-temporal hematoma, sequelar frontal hypodensity of the old stroke with its cranial flap

